# Pioglitazone Upregulates Angiotensin Converting Enzyme 2 Expression in Insulin-Sensitive Tissues in Rats with High-Fat Diet-Induced Nonalcoholic Steatohepatitis

**DOI:** 10.1155/2014/603409

**Published:** 2014-01-14

**Authors:** Wei Zhang, Yi-Zhi Xu, Bo Liu, Rong Wu, Ying-Ying Yang, Xiao-Qiu Xiao, Xia Zhang

**Affiliations:** ^1^Department of Gastroenterology and Hepatology, The Second Affiliated Hospital of Chongqing Medical University, 76 Linjiang Road, Chongqing 400010, China; ^2^Department of Hematology and Oncology, Chongqing the Third Hospital, Chongqing 400014, China; ^3^Institute of Life Sciences, Chongqing Medical University, Chongqing 400016, China

## Abstract

*Background and Aim.* Thiazolidinediones (TZDs) can improve hepatic steatosis in nonalcoholic steatohepatitis (NASH). Angiotensin (Ang) II, the primary effector of renin-angiotensin system (RAS), plays vital roles in the development and progression of NASH. And some AngII-mediated effects can be regulated by TZDs. Angiotensin-converting enzyme (ACE) 2, a new component of RAS, can degrade Ang II to attenuate its subsequent physiological actions. We aimed to evaluate the effects of TZDs on ACE2 expression in insulin-sensitive tissues in NASH rats. *Methods*. Forty rats were divided into the normal control, high-fat diet (HFD), pioglitazone control, and HFD plus pioglitazone groups. After 24 weeks of treatment, we evaluated changes in liver histology and tissue-specific ACE2 expression. *Results*. ACE2 gene and protein expression was significantly greater in liver and adipose tissue in the HFD group compared with normal control group, while was significantly reduced in skeletal muscle. Pioglitazone significantly reduced the degree of hepatic steatosis compared with the HFD group. Pioglitazone significantly increased ACE2 protein expression in liver, adipose tissue, and skeletal muscle compared with the HFD group. *Conclusions*. Pioglitazone improves hepatic steatosis in the rats with HFD-induced NASH and upregulates ACE2 expression in insulin-sensitive tissues.

## 1. Introduction

In conjunction with the growing epidemics of obesity and type 2 diabetes mellitus (T2DM), nonalcoholic fatty liver disease (NAFLD) becomes a common cause of chronic liver diseases in China [1]. NAFLD is a recognized predictor for T2DM, which in turn relates to more severe histological stages of NAFLD [2]. The histological spectrum of NAFLD ranges from simple steatosis, nonalcoholic steatohepatitis (NASH) to NASH-related cirrhosis. Intervention on NASH is the key step to prevent cirrhosis [3]. Due to the close association between NASH and T2DM, antidiabetic drugs may provide a new therapeutic strategy for NASH.

Thiazolidinediones (TZDs), a class of oral antidiabetic drugs, are agonists of peroxisome proliferator-activated receptor (PPAR) *γ*. TZDs, such as rosiglitazone and pioglitazone, have been demonstrated to improve insulin resistance (IR) and hepatic steatosis in NASH [4]. Moreover, clinical observation has shown that TZDs can effectively decrease blood pressure in diabetic patients [5], which involves a crosstalk between PPAR*γ* and renin-angiotensin system (RAS) [6].

RAS is well known for its regulation in hydromineral balance and cardiovascular function. Angiotensin (Ang) II, the primary effector of RAS, has been recognized again for its vital roles in the development and progression of NASH, including increased IR, steatosis, inflammation, and fibrosis [7]. Ang II is formed from Ang I by the catalysing of angiotensin-converting enzyme (ACE). ACE2, a homologue of ACE, degrades Ang II to generate Ang-(1-7) [8]. ACE2 expression influences the Ang II concentration and its subsequent physiological actions [9]. It has been demonstrated that some AngII-mediated effects can be regulated by TZDs [10]. So we hypothesized that the effects of TZDs on NASH may be partially via modulating ACE2.

In addition to the circulating RAS, the concept of local RAS is now recognized. ACE2 has been identified in insulin-sensitive tissues, namely, liver, adipose tissue, and skeletal muscle [11–13]. PPAR*γ* is expressed most abundantly in adipose tissue, followed by liver and skeletal muscle. ACE2 gene expression in adipocytes is upregulated by TZDs [14]. But it remains unclear whether ACE2 expression can be regulated by TZDs in the other insulin-sensitive tissues. For the rats with high-fat diet (HFD) induced NASH, we aimed to detect (1) the changes in tissue-specific ACE2 expression and (2) ACE2 regulation by TZDs in insulin-sensitive tissues.

## 2. Materials and Methods

### 2.1. Animals

Eight-week-old male Sprague-Dawley rats, weighing 110–120 g, were purchased from Experimental Animal Center of Chongqing Medical University and bred in a temperature-controlled (20–22°C) unit with a 12 : 12 light-dark cycle. Forty rats were randomly divided into four groups: (1) a normal control (NC) group (*n* = 10) was fed with the standard chow and gavaged with normal saline; (2) a HFD group (*n* = 10) was fed with the HFD (15 g lard oil and 2 g pure cholesterol were fixed to 83 g standard chow, SLAC Laboratory Animal Co., Ltd., Shanghai, China) and gavaged with normal saline; (3) a pioglitazone control (PC) group (*n* = 10) was fed with the standard chow and gavaged with pioglitazone (10 mg/kg per day; Conba Pharmaceutical Co., Ltd., Zhejiang, China) [15]; and (4) a high-fat diet plus pioglitazone treatment (HP) group (*n* = 10) was fed with the HFD and gavaged with pioglitazone (10 mg/kg per day). The required dose of pioglitazone changed with the daily weight of rats. The care and use of laboratory animals in this study were in accordance with the guidelines approved by the Animal Research Committee of Chongqing Medical University.

### 2.2. Experimental Procedures

All rats were maintained at the above-mentioned conditions for 24 weeks. The blood samples were drawn from the inferior vena cava. Serum or plasma was separated by centrifugation and stored frozen at −80°C. All the rats were executed after 3% sevoflurane anaesthesia. Liver, adipose tissue (epididymal fat), and skeletal muscle (quadriceps muscle) were obtained. One part of these tissues was fixed overnight in buffered formalin (10%) and embedded in paraffin; the other parts were immediately snap-frozen and kept at −80°C until use.

### 2.3. Hepatic Histological Evaluation

Hematoxylin and eosin (H&E) stained sections were scored blindly for the severity of steatosis, lobular inflammation, and hepatocyte ballooning according to the following criteria [16]. For steatosis: grade 0, <5% hepatocytes involved; grade 1, 5%–33% hepatocytes involved; grade 2, 33%–66% hepatocytes involved; grade 3, >66% hepatocytes involved. For lobular inflammation: grade 0, none; grade 1, less than 2 foci per × 200 field; grade 2, 2–4 foci per × 200 field; grade 3, more than 4 foci per × 200 field. For hepatocyte ballooning: grade 0, none; grade 1, few ballooned cells; grade 2, many cells/prominent ballooning.

### 2.4. Biochemical Assays

Fasting plasma glucose (FPG), total cholesterol (TC), high density lipoprotein-cholesterol (HDL-C), low density lipoprotein-cholesterol (LDL-C), triglyceride (TG), free fatty acid (FFA), alanine aminotransferase (ALT), aspartate aminotransferase (AST), and alkaline phosphatase (ALP) concentrations were measured by an autobiochemical analysis apparatus (Hitachi, Tokyo, Japan). Fasting insulin (FINS) concentrations were measured using an insulin radioimmunoassay kit (Beijing Atom High Tech, Beijing, China). Insulin resistance was estimated using the homeostasis model assessment of insulin resistance (HOMA-IR). HOMA-IR = FPG (mmol/L) × FINS (mU/L)/22.5 [17].

### 2.5. mRNA Amplification

Total RNA was extracted from liver, adipose tissue, and skeletal muscle using Trizol reagents (TaKaRa, Dalian, China). Total RNA was quantified by ultraviolet spectroscopy, and reverse transcriptase-polymerase chain reaction assay was performed using ACE2 primer (forward primer: 5′-gac aac ttc ttg aca gcc catc-3′, reverse primer: 5′-acc atc cac ctc cac ttc tcta-3′). *β*-Actin (forward primer: 5′-cct gaa gta ccc cat tga acac-3′, reverse primer: 5′-ctc att gcc gat agt gat gacc-3′) was used as an endogenous control gene for normalization. Amplification conditions for ACE2 and *β*-actin were 5 min at 94°C, followed by 38 cycles of 30 s at 94°C, 30 s at 55°C, and 45 s at 72°C. Each PCR product (5 *μ*L) was subjected to 2% agarose gel electrophoresis and stained with GoldView (Viswagen Biotech, Kerala, India). Each gel was scanned using an imaging system (Bio-Rad, Hercules, CA, USA) and optical density was measured using Quantity One (Bio-Rad Laboratories, California, USA). Experiments were replicated five times.

### 2.6. Western Blot Analysis

Total proteins were extracted from liver, adipose tissue, and skeletal muscle with Radio-Immunoprecipitation Assay (RIPA) lysis buffer (Bioteke, Beijing, China) and separated by sulfate-polyacrylamide gel electrophoresis (SDS-PAGE). Proteins were subsequently transferred to polyvinylidene difluoride membranes (Millipore, Billerica, USA). The blots were blocked with 5% nonfat milk solution for 1.5 h at room temperature and then incubated over night at 4°C with the antibody against ACE2 (Epitomics, California, USA) and incubated at room temperature with peroxidase conjugated goat anti-rabbit IgG secondary antibody (MultiSciences Biotech Co., Ltd., Hangzhou, China). Immunoreactivity was detected by enhanced chemiluminescence detection kit (Keygen, Nanjing, China). The band density of ACE2 was normalized to the corresponding density of *β*-actin (4A Biotech Co., Ltd., Beijing, China).

### 2.7. Statistical Analysis

All data are presented as means ± standard deviation (SD). Differences among multiple groups were compared by one-way analysis of variance (ANOVA), followed by Tukey-Kramer post-hoc test. All calculations were performed with SPSS version 17.0 for Windows (SPSS, Chicago, IL, USA). *P* < .05 was considered statistically significant.

## 3. Results

### 3.1. Liver Weight, Body Weight, and Liver Histology

As shown in Table 1, final body weight, liver weight, and ratio of liver weight to body weight were significantly higher in the HFD group than in the NC group (*P* < .001). Liver weight and ratio of liver weight to body weight were significantly lower in the HP group than in the HFD group (*P* < .01, .001), whereas final body weight did not differ significantly between the HP and HFD groups (*P* > .05).

**Figure 1:**
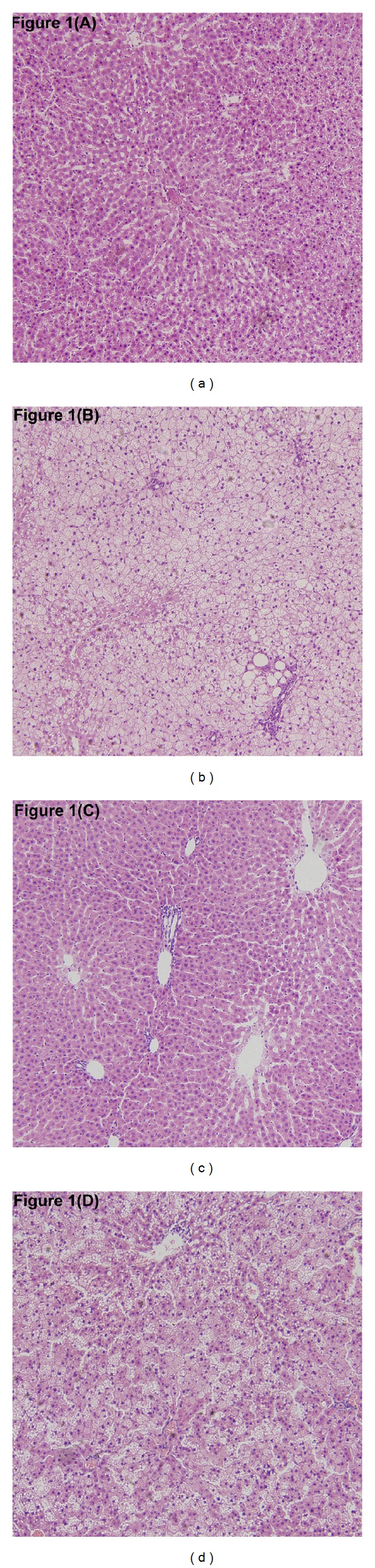
Effects of pioglitazone on hepatic steatosis in rats fed a high-fat diet. Hepatic histopathological changes in the normal control (a) and pioglitazone control (c) groups were within normal limits. Severe hepatic steatosis was found in the high-fat diet group (b). Hepatic steatosis was significantly improved in the high-fat diet plus pioglitazone group (d). (Haematoxylin and eosin; original magnification ×10.)

As shown in Figure 1, macrovesicular steatosis occupied 33% to 66% of the total area (mean score: 2.85 ± 0.23) in the HFD group. On average, there were 2 to 4 foci of lobular inflammation per ×200 field (mean score: 2.25 ± 0.60) and few ballooned cells (mean score: 0.75 ± 0.56) in the HFD group. And significant improvement was observed in steatosis (mean score: 1.35 ± 0.45, *P* < .05), lobular inflammation (mean score: 1.00 ± 0.32, *P* < .05), and hepatocyte ballooning (mean score: 0.35 ± 0.32, *P* < .05) in the HP group compared with the HFD group.

### 3.2. Biochemical Parameters

As shown in Table 1, HOMA-IR (*P* < .001) and concentrations of serum FPG (*P* < .001), FINS (*P* < .001), TC (*P* < .001), HDL-C (*P* < .01), LDL-C (*P* < .001), TG (*P* < .001), FFA (*P* < .001), ALT (*P* < .01), and ALP (*P* < .001) were significantly higher in the HFD group than in the NC group. HOMA-IR (*P* < .001) and concentrations of serum FPG (*P* < .05), FINS (*P* < .001), TC (*P* < .001), HDL-C (*P* < .001), LDL-C (*P* < .001), TG (*P* < .001), FFA (*P* < .001), ALT (*P* < .001), and ALP (*P* < .001) were significantly lower in the HP group than in the HFD group.

### 3.3. Tissue-Specific ACE2 Expression in Insulin-Sensitive Tissues

**Figure 2:**
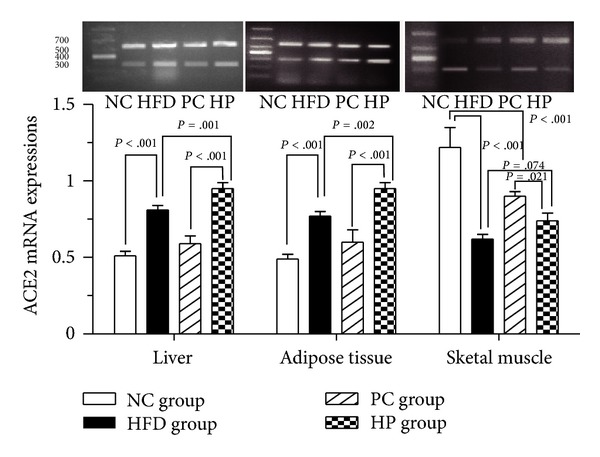
Effects of pioglitazone on ACE2 mRNA expression in insulin-sensitive tissues in rats fed a high-fat diet. In liver and adipose tissue, ACE2 mRNA expression was significantly greater in the HP group compared with the HFD group. In skeletal muscle, ACE2 mRNA expression did not differ between the HFD and HP groups. NC, normal control; HFD, high-fat diet; PC, pioglitazone control; HP, high-fat diet plus pioglitazone.

**Figure 3:**
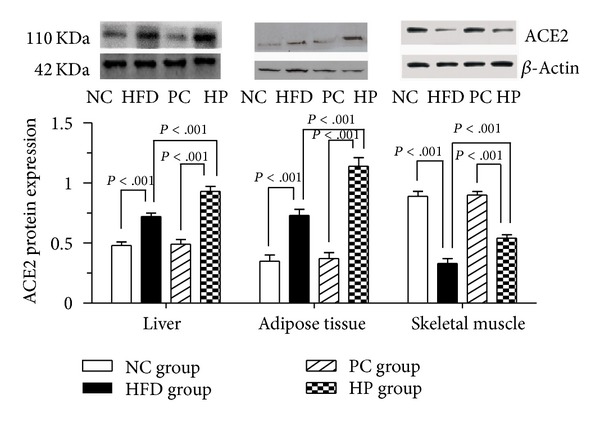
Effects of pioglitazone on ACE2 protein expression in insulin-sensitive tissues in rats fed a high-fat diet. In liver, adipose tissue, and skeletal muscle, ACE2 protein expression was significantly greater in the HP group compared with the HFD group. NC, normal control; HFD, high-fat diet; PC, pioglitazone control; HP, high-fat diet plus pioglitazone.

As shown in Figures 2 and 3, in liver and adipose tissue, ACE2 mRNA and protein expression was significantly greater in the HFD and HP groups compared with the NC and PC groups, respectively. ACE2 mRNA and protein expression was significantly greater in the HP group compared with the HFD group, and no significant differences between the NC and PC groups were observed (*P* > .05). In skeletal muscle, ACE2 mRNA and protein expression in the HFD and HP groups was significantly reduced compared with the NC and PC groups, respectively. ACE2 mRNA expression in the PC group was significantly reduced compared with the NC group. ACE2 mRNA expression did not differ significantly between the HP and HFD groups (*P* > .05), whereas its protein expression was significantly greater in the HP group compared with the HFD group (*P* < .001).

## 4. Discussion

More high-fat food intake with less physical activity is the major etiological factor of NASH. In the present study, we used a HFD-induced NASH model, which has been demonstrated to reproduce the key features of human NASH [18]. After 24 weeks on a HFD, the rats presented with macrovesicular steatosis, lobular inflammation, and hepatocyte ballooning. Accompanied by the histological changes in liver, the increased circulating markers of IR, lipid metabolism, and hepatocellular damage, including FPG, FINS, TC, TG, FFA, ALT, and ALP, were observed in these rats.

In HFD rats, hepatic ACE2 expression increased at the gene and protein levels. Potential mechanism may be that the HFD-induced elevation in FFA activates PPAR*γ* [19], which subsequently regulates RAS [6]. Additionally, the upregulation of hepatic ACE2 expression may result from the NASH-related fibrosis. Previous studies reported that hepatic ACE2 expression increases in both cirrhotic animals and humans [11, 20]. In the present study, HFD rats also presented with an elevation in ACE2 gene as well as protein expression in adipose tissue. It has been reported that the expression of RAS components, including angiotensinogen, renin, aldosterone, and ACE, increases in adipose tissue in animals and humans with obesity-related diseases [21–23], which can be regulated by HFD [24, 25]. Gupte et al. also reported that ACE2 gene expression increases in C57BL/6 mice after 16 weeks on a HFD [14] but not ACE2 protein expression. This may relate to the insufficient feeding time. However in skeletal muscle, HFD rats presented with a reduction in ACE2 gene as well as protein expression. So far, the mechanism contributing to the converse change of ACE2 expression in skeletal muscle remains unclear.

In the present study, pioglitazone improved hepatic steatosis in rats with HFD-induced NASH. And it also decreased HOMA-IR, circulating concentrations of TC, TG, FFA, ALT, and ALP. These findings reinforce the previous data reporting that TZDs lower circulating FFA levels and diminish hepatic fat deposition in diabetic patients [26]. Accompanied by these changes, hepatic ACE2 expression increased at gene and protein levels. The great activation of PPAR*γ* by pioglitazone may contribute to this effect. Circulating and local ACE2 levels can be upregulated by RAS blockers, including angiotensin-converting enzyme inhibitors (ACEIs) and angiotensin II receptor blockers (ARBs) [27, 28]. In our previous study, we have demonstrated that ACEIs can improve insulin resistance and hepatic steatosis in diabetic rats [29]. The similar effects of ARBs have been observed in hypertensive patients by Georgescu et al. [30]. The upregulation of hepatic ACE2 expression may play a key role in the improvement of hepatic steatosis. Interestingly, adipose tissue also exhibited a great elevation in ACE2 expression with the treatment of pioglitazone, parallelled to a high abundance of PPAR*γ* expression. Previous investigation has indicated that the stimulation of PPAR*γ* by rosiglitazone, another agent of TZDs, can increase ACE2 gene expression in 3T3-L1 adipocytes [14]. With the exception of liver and adipose tissue, PPAR*γ* is expressed in skeletal muscle. Similarly, we also found that ACE2 protein expression elevated in skeletal muscle with the treatment of pioglitazone but not ACE2 mRNA expression. The protein expression is not always consistent with mRNA expression, which easily changes with some experimental factors in transcriptional and posttranscriptional procedures.

Previous investigations have demonstrated that PPAR*γ*-regulated gene expression can also influence the function of the components of RAS. PPAR*γ* agonists can attenuate the adverse effects of Ang II by downregulating Ang II concentration and ACE and AT1R gene expression [6]. This is the first study to verify the upregulation of ACE2 by PPAR*γ* agonists in all insulin-sensitive tissues. An elevated ACE2 expression in insulin-sensitive tissues can promote Ang II degrading, attenuating the AngII-induced IR. Several lines of evidence have demonstrated that Ang II promotes IR by impairing the insulin receptors, insulin receptor substrate proteins and the downstream effectors phosphatidylinositol 3′-kinase, protein kinase B, and glucose transporter protein 4 in these insulin-sensitive tissues [31]. Taken together, the tissue-specific ACE2 may be another potential target for improving IR and hepatic steatosis by TZDs.

In conclusion, pioglitazone, one of TZDs, improves hepatic steatosis in the rats with HFD-induced NASH and upregulates ACE2 expression in insulin-sensitive tissues.

## Figures and Tables

**Table 1:** Data for body weight, liver weight, and biochemical parameters in the normal control (NC), high-fat diet (HFD), pioglitazone control (PC), and high-fat diet plus pioglitazone (HP) groups.

	NC group	HFD group	PC group	HP group
Final body weight (g)	449.7 ± 44.7	643.6 ± 127.5***	426.2 ± 34.9	584.9 ± 65.3
Liver weight (g)	14.4 ± 1.4	32.6 ± 8.2***	11.6 ± 1.5	22.5 ± 3.2^††^
Liver/body weight ratio (%)	3.2 ± 0.3	5.0 ± 0.4***	2.7 ± 0.3	3.9 ± 0.6^†††^
FPG (mmol/L)	5.5 ± 0.6	11.8 ± 2.6***	7.3 ± 1.2**	9.8 ± 1.1^†^
FINS (mIU/L)	9.5 ± 1.4	21.6 ± 5.5***	8.0 ± 0.8	14.3 ± 1.9^†††^
HOMA-IR	2.4 ± 0.6	11.3 ± 3.5***	2.6 ± 0.4	6.3 ± 1.2^†††^
TC (mmol/L)	1.9 ± 0.5	3.1 ± 0.5***	1.9 ± 0.2	2.2 ± 0.2^†††^
HDL-C (mmol/L)	1.3 ± 0.2	1.6 ± 0.2***	1.3 ± 0.2	1.2 ± 0.1^†††^
LDL-C (mmol/L)	0.4 ± 0.1	1.5 ± 0.3***	0.5 ± 0.1	1.0 ± 0.2^†††^
TG (mmol/L)	1.1 ± 0.2	2.3 ± 0.3***	0.9 ± 0.2**	1.0 ± 0.2^†††^
FFA (mmol/L)	0.6 ± 0.1	1.0 ± 0.1***	0.7 ± 0.2	0.5 ± 0.1^†††^
ALT (IU/L)	34.3 ± 5.6	91.3 ± 29.1***	31.7 ± 8.7	52.1 ± 4.3^†††^
AST (IU/L)	128.2 ± 49.6	153.4 ± 45.3	126.0 ± 29.9	128.9 ± 20.1
ALP (IU/L)	144.2 ± 43.7	243.0 ± 73.3***	151.0 ± 23.7	163.1 ± 34.4^†††^

Data are presented as mean ± SD. **P < .01, ***P < .001 versus NC group. ^†^ *P* < .05, ^††^ *P* < .01, and ^†††^ *P* < .001 versus HFD group.

FPG: fasting blood glucose; FINS: fasting insulin; HOMA-IR: homeostasis model assessment of insulin resistance; TC: total cholesterol; HDL-C: high density lipoprotein-cholesterol; LDL-C: low density lipoprotein-cholesterol; TG: triglyceride; FFA: free fatty acid; ALT: alanine aminotransferase; AST: aspartate aminotransferase; ALP: alkaline phosphatase.
